# Supplementary material for: Different race pacing strategies among runners covering the 2017 Berlin Marathon under 3 hours and 30 minutes
Source: PLoS One. 2020 Jul 28;15(7):e0236658. doi: 10.1371/journal.pone.0236658 (PMC7386619; doi:10.1371/journal.pone.0236658)
Supplement: Attachment — Submitted filename: Response to reviewers.docx [file pone.0236658.s001.docx]

AUTHORS’ INTRODUCTION

Thank you for providing us with constructive comments regarding our article and for inviting us to submit this revised version. We have made several changes to the manuscript and have addressed all of the comments made by the reviewers. We honestly think that the article has improved as a consequence and is now ready for publication. Thank you very much for your help.

Our reply to the reviewer’s comments can be found below. Our changes in the manuscript are highlighted by track changes.

**REVIEWER 1**

Comments for the Author

Unfortunately, as it stands, I feel the quality of English language used is insufficient to allow publication of the paper in its current format as there are numerous grammatical errors throughout. The work would greatly benefit from detailed proofreading, as there are a lot of places when the meaning of statements is unclear.

Authors’ reply: the English quality of the present manuscript has been improved.

Furthermore (although the findings are slightly surprising & differ from many other analyses in the literature) I dont really see what is novel about this study or what it brings to the body of literature on pacing.

Authors’ reply: the novelty of our study lies in the fact that there are no differences in the race profile between elite and well-training runners when the RP is normalized. This finding is remarkably different compared with previous studies. In turn, women develop less RP variations, as previous studies have showed. However, there are no differences among the best runners and the subsequent group (Top1). This highlight represents a similar race strategy in the elite and well-trained athletes. Another novel point in our study is the comparison among smaller performance groups than previous studies. This has been afforded us to detect differences for women, and no differences, for men, regarding the RP distribution. To sum up, to the best of the author’s knowledge, these results have not yet been shown in other studies.

The rationale is quite 'shaky' and you can’t determine what stategy an athlete 'should' adopt based on a descriptive study of one race such as this.

Authors’ reply: thanks for this feedback. In this sense, the aim of this study has not been to determine the best race strategy based on the runner performance. We completely agree with reviewer’s point of view about this key aspect, it is not possible to decide the best marathon strategy based on a descriptive analysis.

Methodology is generally sound, but the groupings of athletes seems arbitrary and requires justification. for example the >3 hours group in males potentially covers a very wide range of abilities who may have widely varying motivational profiles.

Authors’ reply: we based on the qualifying standard of the World Athletics Championship as an objective way to classify the best runners. After that, we established groups every 15 minutes interval (except for Top1 men group). With the aim of ensuring that the proposed classification reflected the differences in performance between groups, a Kruskal-Wallis test, and subsequent post-hoc test with Bonferroni adjustments, was conducted. This analysis showed that all the groups were significantly different among them.

**REVIEWER 2**

Comments for the Author

Title:
I suggest a more correct (grammar) and precise title: ‘Different race pacing strategies among runners covering the 2017 Berlin Marathon under 3 hours and 30 minutes’.

Authors’ reply: thanks for this suggestion. We have rewritten the title, taking into account the reviewer’s advice.

Abstract:
No reference to the specific race from which data was taken was made.

Authors’ reply: this information has been included in the abstract, lines 4-5.

I also miss some descriptions regarding the specific pacing profiles conducted by both women and men.

Authors’ reply: we completely agree with this point. However, due to the limited extension of the abstract, it has not been possible to include this valuable information in this section.

No reference was made to the data normalization process conducted prior to conducting the different comparisons. This is a critical aspect which should be highlighted. I think that removing some of the specific results found or summarizing them through the indication of ranges rather is needed to include these necessary points suggested.

Authors’ reply: thanks for this note. We have modified the abstract and completed it with the indicated information (lines 8-9).

Introduction:
Authors failed to structure adequately the introduction. In the current study the influence of two main variables (i,e., sex and level of performance) on performance outcomes was measured. Therefore, the introduction should focus on explaining the existing evidence describing the typical pacing strategies observed in elite runners and runners who possesses lower performance level as well as the differences observed by sex. Results from current evidence should be explained according to these aspects.

Authors’ reply: thanks for this feedback. We have improved the introduction, focusing its structure on these two main variables (sex: lines 66 and level of performance: 58).

Methods:

Results:
The table 3 is not clearly indicated. For example, it is not clear which comparisons belong to each split.

Authors’ reply: thanks for this note. We have improved the information regarding table 3 and its description.

Figure 3 and 4 are difficult be understood. For example, no reference to the meaning of X and Y axes is indicated in the legend. It should be clarified.

Authors’ reply: thanks for this note. We have included, in the figure legend, the meaning of “X” and “Y” axes.

Discussion:
From L141 to L165 authors just describe the results found without discussing them according to existing research. This should be part of the results rather than the discussion.

Authors’ reply: we agree with reviewer’s point of view, this subsection of discussion has been discarded.

Referencing swimming studies is not worth in the discussion of a pacing analysis in marathon given the amount of existing pacing studies focused specifically on the marathon race.

Authors’ reply: thanks for this note. We have dismissed these studies and used more suitable ones.

According to the structure suggested for the introduction, authors are encouraged to compare the outcomes observed in the current study separately. For example, comparing the pacing profile observed in elite runners with results observed in existing research focused specifically on elite runners. Same should be done with research focused on runners with lower level of performance. Additionnaly, same should be done when comparing observations made in female runners as some previous research just focused on this specific population. In this sense, I miss the reference to studies strongly related to this specific topic which focused on pacing strategies conducted in marathon runners.

Authors’ reply: thanks for this valuable notice. Taking into account this feedback, several modifications have been conducted in discussion section. Two subsections have been created: Sex per performance differences in pacing and objective profile per sex per performance (lines 177-205).

In addition, when conducting studies of such type, there are some limitations which should be addressed in the text. For example, the influence of running in a group on the pacing strategy selected was not even mentioned. As long as the level of performance is decreasing, the density of runners representing the number of participants running together is also increasing. For example, it is very likely that a higher proportion of runners belonging to the Elite group dropped out from the group they were running with and had to run individually. As the influence of this type of circumstances was not analyzed, this should be stated as a limitation. Hanley (5) studied the influence of running in a group (packing) in elite marathons. In addition, Renfree and Casado reviewed the different existing influences in Athletic races which transform them in complex systems.

Renfree A., Casado A. (2018) Athletics races represent complex systems and pacing behavior should be viewed as emergent phenomenon. Frontiers in Physiology. <https://doi.org/10.3389/fphys.2018.01432>
Furthermore, very likely those runners achieving lower level of performance experienced difficulties to run at a given pace in the beginning of the race as in crowdy races such as Berlin Marathon many runners start the race together and they usually get blocked. This also represents a limitation of this study which should be stated specially when referring to the specific comparison made between groups of different level of performance.

Authors’ reply: thanks for these valuable suggestions. We have included the limitations section considering these useful advices.
