# Supplementary material for: Different race pacing strategies among runners covering the 2017 Berlin Marathon under 3 hours and 30 minutes
Source: PLoS One. 2020 Jul 28;15(7):e0236658. doi: 10.1371/journal.pone.0236658 (PMC7386619; doi:10.1371/journal.pone.0236658)
Supplement: Attachment — Submitted filename: Response to Reviewers.docx [file pone.0236658.s002.docx]

AUTHORS’ INTRODUCTION

Thank you for providing us with constructive comments regarding our article and for inviting us to submit this revised version. We have made several changes to the manuscript and have addressed all of the comments made by the reviewer. We honestly think that the article has improved as a consequence and is now ready for publication. Thank you very much for your help.

Our reply to the reviewer’s comments can be found below. Our changes in the manuscript are highlighted by track changes.

**REVIEWER 1**

Comments for the Author

Abstract:
Line 15 – change to analyse different pacing behaviours, not ‘race paces’ (which will obviously be higher in faster runners). The changes that the reviewer suggests have been made.

Line 22 – change to ‘no statistically significant changes were found…’ The change that the reviewer suggests has been made.

Line 23 – change to ‘a large number of significant differences between splits and groups were found amongst women’. The change that the reviewer suggests has been made.

Line 29-30. Not clear what you mean. We decided to remove the last part of the sentence to improve the understanding.

Line 30-32, As above. Do you mean a positive profile was the most commonly displayed one? We decided to remove the last part of the sentence to improve the understanding.

Introduction:
Line 38 – The marathon is one of the most popular… The change that the reviewer suggests has been made.

Line 41 – delete ‘in the race day’ The change that the reviewer suggests has been made.

Line 44 – replace ‘pointed out’ with ‘demonstrated’ The change that the reviewer suggests has been made.

Line 47-8 – have been implicated in the determination of pacing behavior. The change that the reviewer suggests has been made.

Line 57 – delete ‘a’. The change that the reviewer suggests has been made.

Line 58 – delete ‘the’ before performance. The change that the reviewer suggests has been made.

Line 59 – 60: makes little sense. The sentence has been modified to improve the understanding

Line 62 – change for to by. The change that the reviewer suggests has been made.

Line 64 – change dramatically to dramatic. The change that the reviewer suggests has been made.

Line 66- change the runners to ‘runners’. The change that the reviewer suggests has been made.

Line 67 – change ‘pf the ‘ to ‘towards a’. The change that the reviewer suggests has been made.

Line 71 – change deep speed drop to ‘large speed reduction’. The change that the reviewer suggests has been made.

Line 74 – change ‘main’ to ‘largely’. The change that the reviewer suggests has been made.

Line 75 – what do you mean by ‘broader’? higher? The change that the reviewer suggests has been made.

Line 77 – ‘may be more important in determining…’ The change that the reviewer suggests has made.

Line 78 – change ease to ‘assist’. The change that the reviewer suggests has been made.

Line 81 – change ‘which answers to’ to ‘corresponding to…’ The change that the reviewer suggests has been made.

Line 83 – delete ‘made’. The change that the reviewer suggests has been made.

Line 89 – change ‘making’ to ‘displaying’. The change that the reviewer suggests has been made.

Line 91 – change ‘greater’ to ‘superior’. The change that the reviewer suggests has been made.

Line 95 – change ‘race paces’ to race profiles displayed in the same marathon. The change that the reviewer suggests has been made.

Method
102 – what do you mean by ‘recorded the split’? Sometimes the 5 km splits are not recorded and the organization of the race publishes partial numbers of splits but not all of them. In the present study if a runner did not have every split recorded he/she was excluded from the data analyze.

Line 102 – what is an atypical record? Sometimes the record of the data is wrong. For example, for 5-10 km split it could appear a runner ran at 250% of his average speed, and this is practically impossible.

Line 106 – changed ‘records were obtained’ to ‘ individuals participated’. The change that the reviewer suggests has been made.

Line 106 – change amount to number. The change that the reviewer suggests has been made.

Line 107 - end sentence after info in brackets. The rest is repetition. The change that the reviewer suggests has been made.

Line 109 – ‘into’ 4 groups. The change that the reviewer suggests has been made.

Line 112 – ‘were based on 15 minute time intervals’. The change that the reviewer suggests has been made.

Line 116-117 – As the data was freely available in the public domain, the requirement for informed consent was not necessary. The change that the reviewer suggests has been made.

Line 119 – amended. The change that the reviewer suggests has been made.

Line 122 – change to ‘pacing profile’. The change that the reviewer suggests has been made.

Line 124 – delete ‘percentage’ The change that the reviewer suggests has been made.

Line 125 – delete ‘percentage’ The change that the reviewer suggests has been made.

Line 130 – ‘the marathon for each individual athlete’ The change that the reviewer suggests has been made.

Results:
Line 146 – delete ‘per’. The change that the reviewer suggests has been made.

Line 148 – in men, no statistically significant differences were found… The change that the reviewer suggests has been made.

Line 150 – ‘Amongst women, we observed a large number of differences between groups which were statistically significant’ The change that the reviewer suggests has been made.

Line 153 – change men to male, and ‘the spits within which significant differences were found’.

Line 157 – change practically to almost. The change that the reviewer suggests has been made.

Line 161-162 – change implied to implies and ‘displayed a higher speed relative to mean overall race speed’. The change that the reviewer suggests has been made.

Table 2 – statistically significant but small effect size. Worthy of comment? Yes, we have decided to include a brief commentary about this (line 157-158).

Line 165 – change confirmed to displayed. The change that the reviewer suggests has been made.

Line 168 – change for to ‘by’ , a to ‘the’, and beyond to ‘faster than’. The changes that the reviewer suggests have been made.

Line 169 – ‘a reduction in speed below average…’ The change that the reviewer suggests has been made.

Line 173 – change corroborates to suggests. The change that the reviewer suggests has been made.

Line 174 – change ‘of’ to ‘in’. The change that the reviewer suggests has been made.

Line 176 – delete second ‘per’. The change that the reviewer suggests has been made.

Line 177-8 – Tables 4 and 5 suggest that EP was the most commonly displayed pacing profile in both genders and at all levels of performance. This was followed by PP.’

Delete ‘regarding this’ The change that the reviewer suggests has been made.

Discussion:
Line 185 – we observed no differences in RP… The change that the reviewer suggests has been made.

Line 187 – change showed to demonstrated. The change that the reviewer suggests has been made.

Line 190 – delete ‘per’. The change that the reviewer suggests has been made.

Line 196 – change of to in. The change that the reviewer suggests has been made.

Line 198 – change with to from and which to where. The change that the reviewer suggests has been made.

Line 199 – ‘a previous study by…’. The change that the reviewer suggests has been made.

Line 200 – ‘with EP being the most common profile amongst the best runners’. The

change that the reviewer suggests has been made.

Line 201 – ‘it is important to emphasise that a different course topography can influence observed pacing behaviours’ The change that the reviewer suggests has been made.

Line 204-205 – delete ‘which involved different competition profile’ The change that the reviewer suggests has been made.

Line 205 – the womens elite group was able to maintain a RP above average until … The change that the reviewer suggests has been made.

Line 209 – delete experimented and replace with ‘ experienced a RP drop earlier in the race’ The change that the reviewer suggests has been made.

Line 211 – change develop to display. The change that the reviewer suggests has been made.

Line 214 - showed ‘a similar…’ The change that the reviewer suggests has been made.

Line 217 - in ‘a’ marathon The change that the reviewer suggests has been made.

Line 218 – delete second ‘per’ The change that the reviewer suggests has been made.

Line 221 – the most common profile The change that the reviewer suggests has been made.

Line 225 – an increase in RPE. The change that the reviewer suggests has been made.

Line 228-9 – Diaz et al propose to aim for EP as an optimal strategy. The change that the reviewer suggests has been made.

Line 230-231 – reword this sentence please. This sentence has been remade (line 227-228).

Line 233 – ‘that the majority of runners display EP..’ The change that the reviewer suggests has been made.

Line 237 – St Clair Gibson. The change that the reviewer suggests has been made.

Line 241 – change developed to displayed. The change that the reviewer suggests has been made.

Line 246 – delete ‘because’. The change that the reviewer suggests has been made.

Line 250 – you need to explain this statement. One explanation to this statement has been showed from line 248 to 250.

Line 253 – is there a word missing after ‘establish’? This error has been corrected.

Line 258 – assessed is probably a better word than valued here. The change that the reviewer suggests has been made.

Line 270 – replace ‘on’ with ‘in’. The change that the reviewer suggests has been made.

Line 276 – If shorter splits had been used, we may have found greater variability. The change that the reviewer suggests has been made.

Line 277-8 – ‘we did not consider the effect of running in a group’ The change that the reviewer suggests has been made.

Line 281 - ‘variated’? I do not know this word The change that the reviewer suggests has been made.

Line 282 – change inversely to conversely and ‘may have benefitted from a greater density of runners…’ The change that the reviewer suggests has been made.

Line 291 – ‘well-trained’ and change develop to display. The change that the reviewer suggests has been made.

Line 292 – delete ‘of’. The change that the reviewer suggests has been made.

Line 293 – replace ‘points out’ with ‘indicates’. The change that the reviewer suggests has been made.
